# Traumatic Pulmonary Pseudocysts in a Young Dog Following Non-penetrating Blunt Thoracic Trauma

**DOI:** 10.3389/fvets.2019.00237

**Published:** 2019-07-16

**Authors:** Natosha Mulholland, Iain Keir

**Affiliations:** Department of Emergency and Critical Care, Allegheny Veterinary Emergency Trauma & Specialty, Monroeville, PA, United States

**Keywords:** trauma, pulmonary, pseudocyst, dog, thoracic trauma, traumatic pulmonary pseudocyst

## Abstract

Traumatic pulmonary pseudocysts following non-penetrating blunt thoracic trauma is a well-described phenomenon in the human literature, while in veterinary medicine, this disease process is rarely reported and poorly described in the current literature available. This case report describes a 1.5-year-old male castrated Labrador retriever with findings of pulmonary cysts following a road traffic accident. The goal of this report is to expound upon the pathophysiology, diagnosis, and treatment of this disease process in the veterinary field.

## Introduction

Blunt thoracic trauma manifests in variable clinical entities including pulmonary contusions, lacerations of the pulmonary parenchyma, pneumothorax, hemothorax, and tracheal injury. The development of multiple, thin-walled, air-filled cavities, or traumatic pulmonary pseudocysts (TPPs) is considered rare, occurring in <3% of adults (1, 2) and 4% of children (3) with pulmonary parenchymal injuries. TPPs were first reported by Fallon (4) and have since been reported in human literature in the form of case reports, case studies, and retrospective studies. To the author's knowledge, TPPs secondary to thoracic trauma have rarely been reported in veterinary literature (5). A study by Simpson et al. characterizing the injuries in canine patients following blunt trauma, documented traumatic pulmonary pseudocyst formation in 2% of that patient population.

TPPs form as a result of initial pulmonary parenchymal laceration secondary to blunt thoracic trauma. The laceration then fills with air, fluid or blood resulting in a spherical, thin-walled lesion assumed secondary to elastic chest-wall forces on the surrounding pulmonary tissue. Cyst expansion occurs with ongoing rupture of alveolar walls following initial insult (6). An alternative proposed mechanism of formation is through propagation of concussive waves creating shear stress injury with subsequent tearing of adjacent lung parenchyma (7). These theories were later combined by Khan et al. into a two-step inclusive mechanism (8). In the first step, intrapulmonary pressure is increased following compressive forces from external trauma until the lung parenchyma lacerates. This is followed by decompression of the chest leading to increasingly negative intrathoracic pressure and recoil of the elastic tissue of the lungs. Upon pulmonary parenchymal recoil, small cavities are formed, filling with air or fluid. The cavities grow until the pressure inside is equal to that of the surrounding pulmonary tissue.

TPPs typically appear 24–48 h following injury with a variable clinical presentation ranging from asymptomatic to clinical. Patients may have hemoptysis, coughing or chest pain and can progress to a hypoxemic state potentially requiring mechanical ventilation. Diagnosis in human medicine is often made following computed tomography (CT) of the thorax after patients undergo blunt chest trauma. The prognosis for TPPs is usually good with most patients requiring only supportive care for respiratory dysfunction. Repeat imaging is rarely performed however human literature supports full regression of TPPs after 2–3 months (2, 9).

## Case Summary

A 1.5-year-old, 30.5 kg, cast rated male Labrador presented to a specialty referral hospital after being hit by a car ~1 h prior to arrival. On presentation the patient was ambulatory but weak with mildly tachypnea (55 bpm) and increased inspiratory effort, the mucous membranes were pink and moist with a capillary refill time of 2 s and the heart rate was 120 bpm with synchronous pulses. Oral hemorrhage was present. An approximately 6 cm laceration was appreciated distal to the hock on the medial aspect of the left pelvic limb with moderate discomfort on palpation; no obvious fractures were palpable. Excluding pelvic limb paresis, a complete neurological examination was normal. Methadone (Akron Pharmaceuticals, Lake Forest, Illinois) 0.2 mg/kg was administered IV for pain relief prior to further diagnostics.

**Figure 1:**
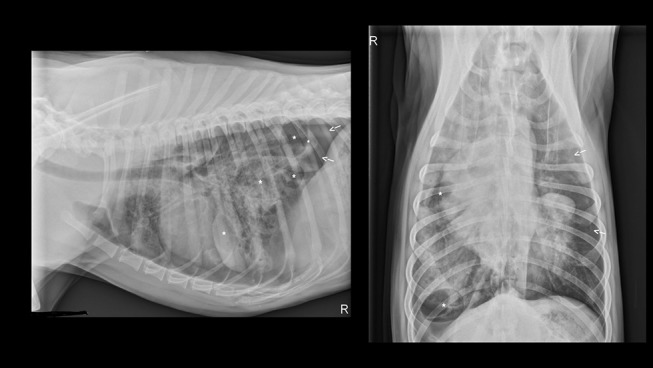
Initial thoracic radiographs performed on presentation to the emergency department following hit by car thoracic trauma. Multiple pulmonary pneumatoceles (indicated by asterisks) and pneumothorax (arrows) appreciated with presence of pulmonary contusions and a mixed interstitial and alveolar pattern.

Abdominal point of care ultrasound (POC US) revealed an intact urinary bladder, no free fluid, consistent with an AFAST Score (AFS) of 0 (10). Right lateral and ventrodorsal radiographs of the thorax and abdomen and dorsoplantar views of the left tarsus were performed. Thoracic radiographs (see Figure 1) revealed a diffuse mild to moderate interstitial lung pattern with ovoid gas lucencies (2.0–4.8 cm) superimposed over the right caudal lung lobes, moderate right-sided pneumothorax, and scant pleural effusion. The entire diaphragm could not be identified clearly on the lateral projection. Soft tissue swelling was present around the tarsus with no overt fracture or luxation present and the abdomen was unremarkable. Blood was collected for a complete blood count, serum biochemistry and venous blood gas/electrolyte analysis. Major abnormalities on serum biochemistry included mild azotemia (BUN 32 mg/dL; RR 25 to 30 mg/dL) and an elevated alkaline phosphatase (736 U/L; RR 10 to 150 U/L). Initial pulse oximetry (SpO_2_) reading was also normal however respiratory rate and effort remained elevated. The patient was hospitalized for further stabilization and supportive care.

A 30 mL/kg fluid bolus of Lactated Ringer's Solution was administered while the patient was prepped for bilateral thoracocentesis. Approximately 800 mL of air was removed from the right hemithorax with an additional 300 mL from the left side. A unilateral nasal cannula was placed, and the patient was started on 1 L/min oxygen supplementation that was later increased to 2 L/min overnight based on SpO_2_ desaturation. The wounds along the medial left tarsus were clipped and sterilely prepped. Sterile staples were used to close the wounds distal to the stifle while a wet-to-dry bandage was placed on the deep laceration for re-evaluation for closure the next day.

The following morning the dog became orthopneic with pale, cyanotic mucous membranes, prolonged capillary refill time and decreased dorsal lung sounds in the left hemithorax. A 20 mL/kg LRS bolus was administered and repeat thoracocentesis of the left hemithorax performed. Approximately 650 mL of air was removed and O_2_ supplementation was increased to 4 L/min. Repeat venous blood gas revealed a decreased hemoglobin (8.0 g/dL, normal 14–20 g/dL) and an elevated lactate (2.7 mmol/L, normal < 2.5 mmol/L). Scant pleural and abdominal effusions were present on repeat POCUS with no other significant findings. Based on the findings of persistent pneumothorax, hemothorax, increasing lactate and initial concern for diaphragmatic hernia on radiographs, a CT scan was performed.

The patient was pre-medicated with hydromorphone (West Ward Pharmaceuticals, Eatontown, New Jersey) 0.1 mg/kg and maropitant (Zoetis Inc, Brazil) 1 mg/kg IV and pre-oxygenated prior to induction of anesthesia with alfaxalone (Alfaxan®, Jurox Pty Limited, Australia) 1.3 mg/kg IV and intubation. The patient was maintained on sevoflurane on 100% O_2_ with routine anesthetic monitoring (ECG, BP, EtCO_2_, Temp, SpO_2_) for the remainder of the CT and post-CT procedures. A CT scan of the thorax was performed using a 4-slice helical scanner with 2 mm slices (Toshiba Aquilon 4, Canon Medical Systems, USA), followed by transfer to the surgical prep area at which time the patient produced ~30 mL of frank hemoptysis from the endotracheal tube and desaturated with an SpO_2_ of 89%. The airway was suctioned and SpO_2_ normalized. Once a level plane of anesthesia was re-established, the right hemithorax was clipped and aseptically prepped. Bilateral thoracostomy tubes were placed as previously described (11). Preservative-free morphine 0.1 mg/kg with 1.5 mg/kg preservative-free bupivacaine (Hospira Inc, Lake Forest, Illinois) was administered into the pleural space for adjunctive analgesia.

**Figure 2:**
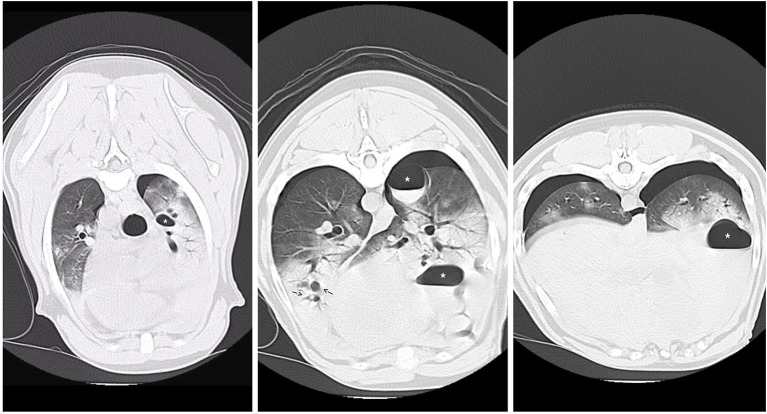
CT images revealing multiple pulmonary blebs (indicated by arrows), pulmonary pneumatocele formation (asterisks) and consolidating alveolar infiltrates within the right cranial and middle lung fields.

CT Scan (see Figure 2) identified multiple contused lungs, a consolidating alveolar infiltrate within the right cranial, middle, and right and left caudal lung lobes, pulmonary blebs and traumatic pulmonary bulla with the largest structure measuring ~6.6 × 3.3 × 3.2 cm and the presence of dependent fluid accumulation consistent with pulmonary hemorrhage. Retraction of the lung lobe margins from the thoracic wall present consistent with pneumothorax and craniodorsal margins of the diaphragm are ill-defined consistent with pleural effusion. All cardiovascular structures, abdominal organs and diaphragm were within normal limits. During recovery from anesthesia, the patient de-saturated when initially placed back on nasal cannula oxygen flow but improved when the flow rate increased to 8 L/min and he recovered without any further complications. Continued tube care initiated with intermittent thoracic drainage tube aspiration every 4 h. Analgesia was provided by hydromorphone (West Ward Pharmaceuticals, Eatontown, New Jersey) 0.1 mg/kg IV every 4–6 h in addition intrapleural analgesia was provided by bupivacaine (Hospira Inc, Lake Forest, Illinois) 1.5 mg/kg Q24H and hydromorphone (West Ward Pharmaceuticals, Eatontown, New Jersey) 0.1 mg/kg Q6H via chest tube. Respiratory monitoring continued via serial arterial blood gas and SpO_2_ monitoring. A normalized creatinine was appreciated via repeat biochemistry testing 24 h following presentation. The pelvic limb bandage was changed every 48 h.

**Figure 3:**
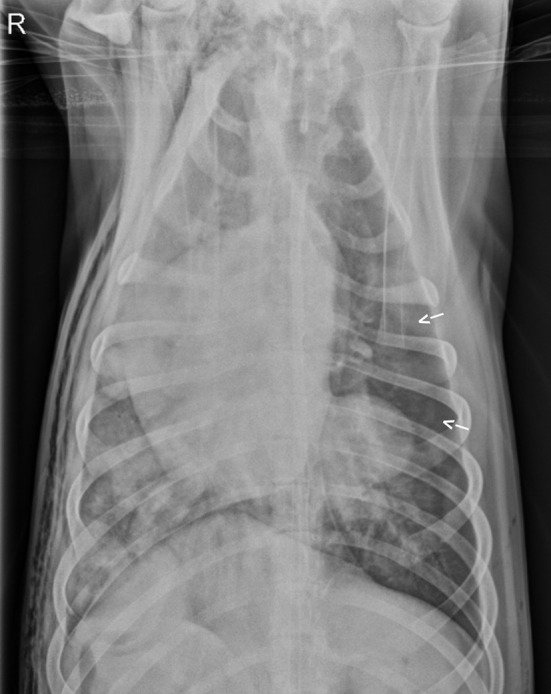
Repeat ventrodorsal thoracic radiographs following thoracostomy tube removal. There is marked improvement in pulmonary contusions with persistence of a heavy coalescing mixed interstitial and alveolar pattern persisting diffusely in the right hemithorax. Pneumothorax (arrows) also persistent.

Three days after hospital admit, minimal blood and air were able to be aspirated from the thoracostomy tubes (<2 mL/kg/day) and both tubes were removed. On day four, supplemental oxygen via nasal cannulas was discontinued based on a normal SpO_2_ off oxygen supplementation (see Table 1) and appropriate respiratory rate and effort on a room air trial. Repeat ventrodorsal thoracic radiographs revealed marked improvement in pulmonary contusions with a heavy coalescing mixed interstitial and alveolar pattern persisting diffusely in the right hemithorax (see Figure 3). A round 5 ×5 cm soft tissue opacity was visible in the left hemithorax consistent with hemorrhage filled traumatic pulmonary pseudocyst. The dog was bright, eating, and comfortable after transitioning to oral medications (Codeine 60 mg PO Q6H, Amoxicillin Clavulanic Acid 500 mg PO Q12H, Carprofen 50 mg PO Q12H) and was discharged that evening. Follow up with owners at ~2 and 6 months after discharge revealed the patient was doing well and experiencing no respiratory complications. As the patient was clinically normal at the time of follow up, the owners declined further diagnostic imaging.

**Table 1:** Overview regarding oxygen parameters at different times during hospitalization.

	**Day 1**	**Day 2**	**Day 3**	**Day 4**	**Day 5**
SpO_2_	_RMAIR_ 89% _2LPM._ 96%	_RMAIR_ 79% _2LPM_ 89% _4LPM._ 95%	_RMAIR_ 90% _8LPM_ 96%	_RMAIR_ 92% _4LPM_ 95%	_RMAIR_ 94%
PO_2_ (_ARTERIAL_)			45.3 g/dL		
PCo_2_ (_arterial_)			39.6 mmHg		
Pco_2_ (_venous_)	39 mmHg	45.2 mmHg	53.4 mmHg		

## Discussion

Traumatic pulmonary pseudocyst formation following blunt force trauma is suggested to occur in about 2% of veterinary patients, however the pathophysiology and clinical management of TPP's is poorly described in the veterinary literature. TPP's are a well-documented phenomenon in human literature. From the initial description in 1940 through to 2018, numerous case reports, case series and retrospective studies have been published in human journals detailing the pathophysiology of the disease, medical management, needs for ventilatory or surgical intervention, common sequelae of cyst formation and histopathological descriptions.

Simpson et al. (12) describes the presence of traumatic bullae formation in 2% (4/235) of a patient population presenting through the ICU with evidence of pulmonary bullae formation. While the idea of pseudocyst formation following trauma is not novel, this number would suggest that it is an uncommon manifestation following blunt thoracic injury. This paper does not describe how the diagnosis of bullae was made, the pathophysiology of cyst formation, in-hospital treatment or long-term outcome of the patients with bullae formation.

In slight contrast to the numbers reported in the Simpson study, a 1999 study by Powell et al. evaluated a population of patients following motor vehicle trauma with confirmed pulmonary contusions (13). In the reported population, 10% (14/143) of patients had evidence of pulmonary pseudocyst formation. The author's postulated contributing factor to the increased prevalence of bulla formation in this patient population is the presence of pre-existing contusions.

A recent case report describing pseudocyst formation was published by Barge et al. (5). The case report describes a young dog presenting to the ICU 3 days post road traffic accident. A tentative diagnosis of TPPs was made via thoracic radiographs on the day of presentation. On CT scan 3 days later, the presence of TPPs was confirmed. In contrast to the patient described by the current case report, the patient described by Barge et al. received percutaneous drainage of the largest cavity visible on CT scan. The patient also received supportive care with oxygen supplementation and all signs of respiratory distress and hypoxemia resolved without surgical intervention.

As previously discussed, traumatic pulmonary pseudocysts (TPPs) occur following blunt thoracic injury. Different nomenclature has been used over the years to describe trauma induced pulmonary cysts including cavitary pulmonary lesions, pseudocystic hematomas, traumatic lung cavities and traumatic cysts (14–16). The term traumatic pulmonary pseudocyst (TPP) has been adopted and used most commonly in human literature as it indicates a structure absent of an epithelial lining. This is important as cysts without epithelial lining are easily absorbed and considered more benign (6). In veterinary literature the terms traumatic bullae, pulmonary pneumatocele and hemopneumatocele have been used (5, 12, 17).

Traumatic parenchymal pseudocysts have been further classified into four categories by Wagner et al. (18). Type 1 include air or air-fluid filled structures visible on CT scan resulting from sudden compression of the chest wall where the lung ruptures. With Type 2, the cavity is visible within the paravertebral lung occurring when more pliable lower chest wall is acutely compressed and displacement of the lower lobe across a vertebral body, leading to shearing type injury. Type 3 are small peripheral cavity or linear radiolucency that is always close to the chest wall with visible rib fracture and puncturing of the lung. Type 4 results from previously formed pleuropulmonary adhesions leading to lung tearing with thoracic chest trauma which are visible only at autopsy or during surgery. According to Wagner, out of 75 people with a total of 115 lacerations following blunt chest trauma, Type 1 and Type 3 injuries occurred most commonly. To the author's knowledge, no classification system has been utilized in veterinary medicine to date.

In human literature TPPs occur most commonly in people < 30 years old, however, there are case reports of patients 30 years or older with pseudocyst formation following blunt thoracic trauma in circulation (2, 19–21). Age predilection is assumed secondary to increased thoracic wall pliability in young adults leading to greater transmission of force through the chest leading to pseudocyst formation (2, 9, 22). The patient in this study is 1.5 years old and would be classified as a young adult. Vehicle trauma has been reported as the leading cause for TPP formation in many studies with those individuals being struck by a vehicle having a higher incident of TPP (9). The patient in this study falls within the latter group making the development of pseudocysts a plausible side effect of patients who are involved in motor vehicle accidents.

Traumatic pulmonary pseudocysts are most commonly diagnosed through the use of thoracic computed tomography as radiographs are less likely to be diagnostics for TPPs (23). A 1989 study showed that TPPs are not visible on plan thoracic radiographs (TXR) until 3 to 14 days post-injury (24). This is supported in a 2003 study in which chest radiographs obtained on the day of injury in 10 patients following blunt thoracic trauma did not show any radiologic sign of pseudocysts. These 10 patients underwent CT scan on day 1 (8 pts) or day 2 (2 pts) where TPP was diagnosed in all cases (2). A more recent 2017 paper investigating pediatric blunt trauma in patients ranging from age 3 to 17 found that TXR was only successful in diagnosing TPP in 4 out of 25 patients diagnosed with pulmonary pseudocysts while CT was successful in identifying TPP in all cases. In the previously published veterinary case report, the dog was also a young adult and evidence of TPP was seen on initial thoracic radiographs at admit. This finding was assumed secondary to the patient presenting 3 days post HBC (5). In the dog reported in this study, lesions were identified on initial radiographs performed at admission. This may be secondary to the severity of impact that this patient endured or difference in time to development of TPPs in the canine species. However, given the patient's age, the presence of congenital pseudocysts or emphysema cannot be ruled out entirely.

## Concluding Remarks

This study supports traumatic pulmonary pneumatoceles as a differential diagnosis for canine patients undergoing non-penetrating blunt thoracic trauma. TPPs can be amenable to conservative medical management without the need for surgical intervention and resolution often occurs within weeks to months after initial injury. Further investigations would be warranted to determine efficacy of CT vs. TXR in diagnosing TPPs in the veterinary patient and the long-term implications of these findings.

## Consent

Informed consent was obtained from owners prior to the writing and submission of this case report.

## Author Contributions

NM assisted in primary case management and wrote the manuscript. IK supervised the clinical management of the case. All authors critically reviewed and approved the final version of the manuscript.

### Conflict of Interest Statement

The authors declare that the research was conducted in the absence of any commercial or financial relationships that could be construed as a potential conflict of interest.
